# Short Cut to Eugenics

**Published:** 1915-12

**Authors:** 


### Short Cut to Eugenics.

Even eugenics has not escaped the tendency of the times to circumvert the real problems of life. We are all used to short cuts in acquiring language, commercial problems, etc., and even in mental efficiency, and now comes a short cut to bettering the race in one generation. Why take "an hundred years to make a man," as Dr. Holmes once said it would, when he can be made in nine months, by simply adhering to prenatal culture? We have long been familiar with the idea of maternal impressions, but it is only recently that some so-called eugenic writers have advanced this idea as a means to eugenic reform. It would be like the millennium indeed, were it true that a superior woman could marry a profligate man and by mental willing and healthful deportment she could insure her offspring to be as she desired. If great orators or singers or mechanics could be made by prenatal culture; if character and temperaments could be formed by right living during the embryonic life of the child, then the whole question of eugenics reform would rest in inducing prospective mothers to live in a manner to assure this desired end.

So-called examples of maternal impressions are simply coincidence and not causation. That any possible influence could be exerted on the child is scientifically impossible, as there is no physical connection existing between mother and child. The child develops its own body out of the potentialities received in the germ plasm of the parents, and receives its nournishment through the placental circulation. It is true nutrition may be affected by the physical condition of the mother, and in this way result in a low development of the child. Thus a child at its birth may be poorly nourished, or its organs may be poorly developed. Whatever effects this general nutrition of the mother; e. g., bad air, overwork, under nourishment, worry and nerve strain, will be felt in the immaturity of the child. But talents, temperaments, habits and physical marks are impossible to transfix.

What is so often taken for maternal impressions is the coincidence of a hereditary strain existing at the same time in the mother; e. g., a woman who displays a degree of temper, or who so worries that her digestion is impaired, will affect her own nutrition and likewise that of the child. If, perchance, the child develops a cross, peevish disposition, she attributes this fact to her temperament or her worrying, when, in fact, the child has simply inherited the mother's disposition. These coincidences are very much like the superstitions regarding making trips on Friday. If the accident should occur, it is attributed to the fatal day, but no explanation is made of why they do not occur when the day is uneventful or why they are just as frequent on any other day.

It should be remembered, too, that the so-called maternal impressions always occur after the child has fully developed its features; i. e., by the end of the second month, a time when few mothers are aware that they are pregnant. Many causes may arrest the development of the embryo at any stage, but these are chiefly embryonic and, perhaps, accounts for the physical defects sometimes encountered and which are attributed to mental impressions.

With education superstitions are being eradicated, but with some, such as this, the foundation is so deeply rooted by social heredity that it takes the process of evolution to stamp it out. Until this is done, teachers of eugenics will have to steer clear of the "short cuts" and learn the old, old lesson that nothing lasting is ever accomplished save by the hard and slow process of diligence and patience.

